# *Aloe barbadensis* Mill. extract improves symptoms in IBS patients with diarrhoea: post hoc analysis of two randomized double-blind controlled studies

**DOI:** 10.1177/17562848211048133

**Published:** 2021-10-08

**Authors:** Bani Ahluwalia, Maria K. Magnusson, Lena Böhn, Stine Störsrud, Fredrik Larsson, Lena Öhman, Magnus Simrén

**Affiliations:** Department of Microbiology and Immunology, Institute of Biomedicine, University of Gothenburg, Gothenburg, Sweden; Research and Development, Calmino Group AB, Gothenburg, Sweden; Department of Microbiology and Immunology, Institute of Biomedicine, University of Gothenburg, Gothenburg, Sweden; Research and Development, Calmino Group AB, Gothenburg, Sweden; Department of Molecular and Clinical Medicine, Institute of Medicine, University of Gothenburg, Gothenburg, Sweden; Department of Molecular and Clinical Medicine, Institute of Medicine, University of Gothenburg, Gothenburg, Sweden; Research and Development, Calmino Group AB, Gothenburg, Sweden; Department of Microbiology and Immunology, Institute of Biomedicine, University of Gothenburg, Box 435, 405 30 Gothenburg, Sweden; Department of Molecular and Clinical Medicine, Institute of Medicine, University of Gothenburg, Gothenburg, Sweden; Center for Functional Gastrointestinal & Motility Disorders, School of Medicine, The University of North Carolina at Chapel Hill, Chapel Hill, NC, USA

**Keywords:** Aloe extract, diarrhoea, gastrointestinal symptoms, IBS-D, irritable bowel syndrome

## Abstract

**Background:**

*Aloe barbadensis* Mill. (Aloe) extract was found to be well-tolerated, safe and showed beneficial effects in subsets of irritable bowel syndrome (IBS) patients in two randomized, double-blind, controlled studies. However, the individual studies were underpowered to perform subgroup analyses. We therefore determined the effect of Aloe extract in IBS subgroups in a post hoc analysis combining the results from the two studies.

**Methods:**

Data from the two controlled studies comparing Aloe and control treatment taken orally for 4 weeks, were pooled. Both studies included IBS patients fulfilling the ROME III criteria and IBS Symptom Severity Score (IBS-SSS) was assessed. We analysed the effect of Aloe extract on IBS symptom severity and the proportion of responders (IBS-SSS reduction ⩾ 50) in IBS subgroups.

**Results:**

In total, 213 IBS patients were included in the post hoc subgroup analyses. A reduction in overall symptom severity, primarily driven by effect on pain severity and frequency, comparing baseline versus end of treatment, was recorded in IBS patients with diarrhoea (IBS-D) receiving Aloe (*n* = 38, *p* < 0.001) but not control treatment (*n* = 33, *p* = 0.33), with difference between the treatment groups (*p* = 0.01). Moreover, the frequency of responders was higher in IBS-D patients receiving Aloe (*n* = 22, 58%) compared to control treatment (*n* = 10, 30%) (*p* = 0.02). The effect of Aloe extract treatment on IBS symptom severity was not superior to control treatment in the other IBS subtypes.

**Conclusion:**

Aloe extract improves symptom severity in IBS-D patients and can be regarded as a safe and effective treatment option for this patient group.

## Introduction

Irritable bowel syndrome (IBS) is a chronic and highly prevalent disorder of a disturbed brain-gut interaction characterized by recurrent abdominal pain and disturbed bowel function.^1,2^ Despite much efforts, the underlying pathophysiology of IBS is still poorly understood.^3^ IBS patients can be categorized into four distinct subtypes according to the ROME criteria which are based on predominant stool pattern: IBS with diarrhoea (IBS-D), constipation (IBS-C), mixed (IBS-M) and an undefined pattern of abnormal stool (IBS-U).^1^ However, stool patterns vary considerably among patients with the same subtype and a majority of the patients switch between the bowel habit subtype over time.^4^ As a consequence of this complexity and heterogeneity, the current treatment options for IBS, which predominantly target the individual symptoms, are limited and make adequate treatment of global IBS symptoms a significant challenge.

Limitations in the current available treatment options for IBS have led to the popularity of complementary and alternative therapies,^5,6^ despite the often poor clinical evidence supporting their efficacy.^7,8^ *Aloe barbadensis* Mill. (Aloe) being a medicinal plant, well reputed for its diverse therapeutic applications,^9,10^ is a commonly used alternate therapy for alleviating IBS symptoms. While some previous clinical studies have demonstrated beneficial effects of Aloe in improving IBS symptoms,^11–14^ the evidence is still contradictory and incomplete.^11,15^

In two randomized controlled studies performed by our group, using the same extract, Aloe was found to be well tolerated and safe in IBS patients and showed tendency to improve symptom severity in subsets of IBS patients.^13,14^ However, the individual studies were underpowered to carry out subgroup analyses and confirm Aloe treatment effects in IBS subtypes. To address this, we conducted a post hoc analysis of pooled data from the two randomized controlled studies. The main objectives were to determine the effect of Aloe treatment on overall symptom severity relative to baseline and the proportion of responders in IBS subtypes based on predominant bowel habit. Treatment effects of Aloe on other IBS subgroups based on IBS severity and psychological distress were also evaluated.

## Material and methods

### Study cohort and study design

This post hoc analysis is based on previously reported data from two randomized, controlled trials (NCT01400048),^13,14^ hereafter mentioned as Study A and Study B, respectively. The study cohort and study design for both trials have previously been described in detail, and the study design including the inclusion and exclusion criteria were the same in both studies.^13,14^ Briefly, adult patients (18–70 years old) meeting the Rome III criteria for IBS^16^ were recruited at the gastroenterology outpatient clinic of Sahlgrenska University Hospital, Gothenburg. Study patients were randomized to Aloe treatment receiving Aloe extract (Calmino group AB) effervescent tablets (500 mg Aloe and 780 mg inulin/day) or control treatment effervescent tablets (1280 mg inulin/day), both treatment tablets having similar taste, respectively for 4 weeks. The Aloe extract used in the treatment tablets was a freeze-dried inner leaf gel extract with a complex polysaccharide content containing less than 0.1 ppm aloin (anthraquinone), a component of Aloe known for its laxative properties.^9^

Eligible study patients completed questionnaires to characterize their symptom severity and bowel habits. IBS Severity Scoring System (IBS-SSS),^17^ a 2-week stool diary based on Bristol stool form (BSF) scale^18^ and the Hospital Anxiety and Depression (HAD) scale^19^ (see supporting information for more detail) were completed at baseline and at end of treatment. All patients who agreed to participate in the studies were given verbal and written information before giving their written consent to participate in the study. The study protocol was approved by the Regional Ethical Review Board in Gothenburg (DNR: 365-07).

### Subgroup characterization of study subjects

Classification into IBS subtypes according to the Rome III criteria was performed based on Bristol Stool Form scale characteristics: IBS with constipation (IBS-C), IBS with diarrhoea (IBS-D), IBS patients with mixed bowel habits or mixed IBS (IBS-M) and unsubtyped IBS (IBS-U).^16^ IBS-M and IBS-U were combined into one group (IBS-nonCnonD). IBS severity subgroups were based on validated cut-off scores for IBS-SSS: mild IBS, IBS-SSS score < 175; moderate IBS, IBS-SSS score of 175–300; and severe IBS, IBS-SSS score > 300.^17^ Furthermore, IBS subgroups according to different levels of psychological distress were defined based on validated cut-off scores for HAD scale. HAD score ⩾ 8, including both borderline and clinically significant cases, defined IBS patients with anxiety or depression, respectively, and HAD score < 8, patients without anxiety or depression, respectively.^19^

### Study outcome and data analyses

For this post hoc analysis, the primary outcome was the change in IBS-SSS at the end of the treatment period relative to baseline and the proportion of responders in the various subgroups based on IBS subtypes, severity groups and patients with or without psychological distress. A responder was defined as a patient with a reduction in IBS-SSS ⩾ 50 points, which is considered to reflect a clinically meaningful improvement, at the end of the treatment period relative to baseline.^17^ As secondary endpoints, we analysed the effect of Aloe treatment and control treatment on the individual sub-scores of the IBS-SSS, bowel habits measured by BSF^16^ and HAD, both within-group and between group differences. All analyses were performed on pooled data from patients included in the per-protocol analysed population, that is, not including drop-outs from the two studies.

### Statistical analyses

All analyses were based on pooled results from the two controlled trials unless otherwise stated. Univariate statistical analysis was performed using SPSS statistical package, V.24.0 (Released 2016, IBM Corp, Armonk, NY, USA). Categorical variables were compared using the χ2 test, and continuous variables were compared using either the independent samples and paired samples *t*-test or the corresponding non-parametric Mann–Whitney U test and Wilcoxon signed rank text, based on normality of distribution, demonstrated with histograms and Shapiro-Wilk statistic test.

Generalized linear model (with logit link function) using response as the binary variable, was used to evaluate treatment response in relation to baseline explanatory variables (GLM logistic function in SPSS statistical package, V.24.0). Generalized liner model analysis estimated variables associated with response (*p* < 0.05) as well as the odds ratios (OR) for being a responder. The odds ratio was converted to relative risk ratio (RR) using the following formula $\hat{RR}=\frac{odds\;ratio}{\left(1-risk_{0}\right)+(risk_{0}\times odds\;ratio)}$ where risk_0_ is the risk of having the outcome in the control group.^20^

Data in text, figures and tables are shown, corresponding to the statistical test used, as mean (SD) or median followed by 25th and 75th percentile, respectively. *p* values < 0.05 were considered statistically significant.

## Results

### Analysis population

Demographic and baseline characteristics of patients in the individual studies were similar with regards to age, sex and baseline symptom severity and hence the two studies were deemed acceptable for pooling (Table 1). The IBS subtypes (IBS-C, IBS-D and IBS-nonCnonD) although randomized and represented in both studies, were unequally distributed between the two studies (*p* = 0.02) (Table 1). The pooled post hoc analysis, comprised a total of 223 patients, where 116 were in the Aloe treatment group and 107 in the control group (Table 2). Demographics and baseline characteristics in the pooled analysis were similar between the two treatment groups in regards to age, sex and distribution of patients in the various subgroups. However, the Aloe treatment group had a higher baseline symptom severity compared to the control group (*p* = 0.03) (Table 2). Dropouts from the two studies (supplemental Table S1), were not included in this post hoc analysis, however based on the limited or no adverse effects previously reported in the individual studies,^13,14^ Aloe treatment was well tolerated and safe in IBS patients.

**Table 1.** Clinical and Demographic Characteristics for IBS patients in Study A and Study B.

Baseline characteristics	Study A (*n* = 63)	Study B (*n* = 160)	*p* value
Gender, F/M (%)	46/17 (73/27)	128/32 (80/20)	0.26
Age, years, median (interquartile range)	45 (32–56)	45 (31–56)	0.85
IBS-SSS, mean ± SD	293 ± 88	283 ± 95	0.48
IBS symptom severity based on IBS-SSS during screening, number of patients			0.61
Mild (75–175)	6	23	
Moderate (175–300)	26	65	
Severe (>300)	31	72	
IBS subtypes based on predominant bowel habits during screening, number of patients			*0.02*
IBS-C	19	47	
IBS-D	28	43	
IBS-nonCnonD	16	70	
Hospital Anxiety and Depression Scale at screening, median (interquartile range)			
Anxiety	5 (3–9)	5 (3–10)	0.51
Depression	4 (1–7)	3 (1–5)	0.09

Significant *p* values are displayed in italics.

IBS, irritable bowel syndrome; IBS-C, IBS with constipation; IBS-D, IBS with diarrhoea; IBS-nonCnonD, mixed-type IBS and unsubtyped IBS; IBS-SSS, IBS Symptom Severity Score.

**Table 2.** Clinical and demographic characteristics of pooled post hoc study population.

Baseline Characteristics	Aloe group (*n* = 116)	Control group (*n* = 107)	*p* value
Gender, F/M (%)	94/22 (81/19)	80/27 (75/25)	0.26
Age, years, median (interquartile range)	44 (31–57)	46 (33–55)	0.63
IBS-SSS, mean ± SD	299 ± 87	272 ± 97	*0.03*
IBS symptom severity based on IBS-SSS during screening, number of patients			0.053
Mild (75–175)	9	20	
Moderate (175–300)	50	41	
Severe (>300)	57	46	
IBS subtype based on predominant bowel habits during screening, number of patients			0.92
IBS-C	33	33	
IBS-D	38	33	
IBS-nonCnonD	45	41	
Hospital Anxiety and Depression Scale at screening, median (interquartile range)			
Anxiety	5 (3–10)	5 (3–9)	0.73
Depression	3.5 (1–6)	3 (1–6)	0.24

Significant *p* values are displayed in italics. IBS, irritable bowel syndrome; IBS-C, IBS with constipation; IBS-D, IBS with diarrhoea; IBS-nonCnonD, mixed-type IBS and unsubtyped IBS; IBS-SSS, IBS Symptom Severity Score.

### Aloe extract improves symptoms in IBS patients with diarrhoea

**Figure 1.**
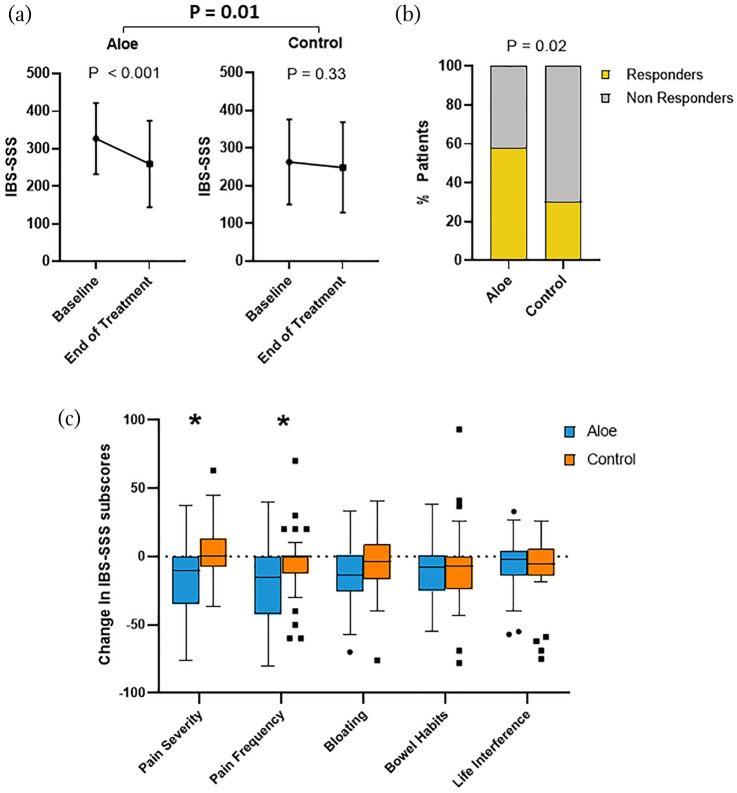
Effect of Aloe and control treatment on severity of total gastrointestinal symptoms and overall response in IBS patients with diarrhoea. (a) IBS symptom severity, measured by IBS-SSS (mean ± SD), at baseline and end of the treatment in patients in both treatment groups. (b) Proportion of responders in both treatment groups, i.e. number of patients with a reduction of IBS-SSS ⩾ 50 points at the end of the treatment vs baseline. (c) Boxplot depicting changes in IBS-SSS subscores at the end of treatment period relative to baseline. Asterisks represent significant *p* values: * < 0.05. IBS-SSS, IBS Symptom Severity Scale.

**Figure 2.**
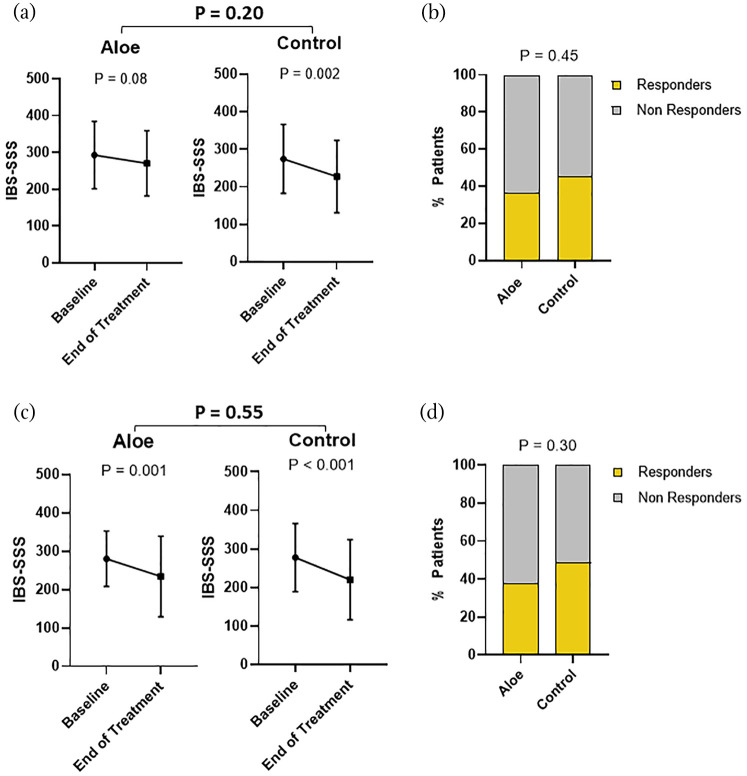
Effect of Aloe and control treatment on severity of total gastrointestinal symptoms and overall response in IBS patients with constipation (IBS-C) and mixed and unsubtyped IBS (IBS-nonCnonD). IBS symptom severity, measured by IBS-SSS (mean ± SD), at baseline and end of the treatment in both treatment groups seen in IBS-C patients (a) and IBS-nonCnonD patients (c). Proportion of responders in both treatment groups, i.e. number of patients with a reduction of IBS-SSS ⩾ 50 points at the end of the treatment vs baseline in the IBS-C (b) and IBS-nonCnonD subtype (D).

We first analysed the effect of the two treatment groups on IBS subtypes based on predominant bowel habits. A reduction in overall symptom severity, comparing baseline versus end of treatment, was recorded in IBS-D patients receiving Aloe (*p* < 0.001) but not control treatment (*p* = 0.33), with a difference between the treatment groups (*p* = 0.01) (Figure 1(a), Table 3). Moreover, the frequency of responders was higher in IBS-D patients receiving Aloe (*n* = 22, 58%) compared to control treatment (*n* = 10, 30%) (*p* = 0.02) (Figure 1(b)). The effect of Aloe treatment on IBS symptom severity was, however, not superior to control treatment in the other IBS subtypes (IBS-C and IBS-nonCnonD) based on predominant bowel habit (Table 3, Figure 2).

**Table 3.** Effects on IBS symptoms in IBS subtypes based on bowel habit (pooled data).

	Aloe group				Control group				*p* value between treatment groups^a^
	n	Baseline	End of treatment	*p* value within group	n	Baseline	End of treatment	*p* value within group	
IBS-SSS total score (IBS-D)	38	327 ± 94	260 ± 115	*<0.001*	33	263 ± 113	249 ± 120	0.33	*0.01*
IBS-SSS total score (IBS-C)	33	293 ± 92	270 ± 89	0.08	33	274 ± 92	227 ± 96	*0.002*	0.20
IBS-SSS total score (IBS-nonCnonD)	45	280 ± 72	234 ± 105	*0.001*	41	277 ± 88	220 ± 104	<0.001	0.55

Data shown as mean ± SD. Significant *p* values are displayed in italics. IBS, irritable bowel syndrome; IBS-C, IBS with constipation; IBS-D, IBS with diarrhoea; IBS-nonCnonD, mixed-type IBS and unsubtyped IBS; IBS-SSS, IBS Symptom Severity Score.

a Based on change in IBS-SSS total score, comparing baseline vs end of treatment.

Further, when assessing the effect of the two treatments on the individual items of the IBS-SSS score, the greater overall reduction in symptom severity seen in the IBS-D group receiving Aloe vs control treatment was primarily driven by its effect on pain severity and pain frequency (Figure 1C). Both treatment groups had similar effect on stool consistency (Aloe: 5.5 (5–6) vs 4.9 (4–6); *p* = 0.005; control: 5.4 (5– 6) vs 4.9 (4 – 6); *p* = 0.045) and stool frequency (Aloe: 2.5 (1.3–3) vs 2.3(1.4–2.9); *p* = 0.29; control: 2.6 (1.7–3.1) vs 2.5 (1.4–3.4); *p* = 0.4), with no differences between the treatment groups.

### Predictors for treatment response in IBS patients with diarrhoea

When comparing responders and non-responders among the IBS-D patients, the χ2 tests of homogeneity for response *vs* symptom severity at baseline (defined by IBS-SSS: mild, moderate, severe) showed no evidence for response being linked to more severe symptoms before intervention for the Aloe group (*p* = 0.20) or the control group (*p* = 0.57). However, the treatment response seen in the IBS-D group showed an association with gender, with increased frequency of responders seen among females (Aloe responders; *n* = 17 (65%) and control responders; *n* = 10 (45%)) as compared with males (Aloe responders; *n* = 5 (42%) and control responders; *n* = 0 (0%)) (*p* = 0.004).

Given the association between treatment response and gender and the fact that IBS-D patients receiving the Aloe treatment had a higher baseline symptom severity compared to those in the control group (327 ± 94 vs 263 ± 113, *p* = 0.01), a generalized linear model corrected for baseline symptom severity and gender was performed to determine association between response and treatment group among IBS-D patients. The odds for IBS-D patients treated with Aloe of becoming a responder were over three times higher (OR: 3.3; CI: 1.08–9.98) than for patients in the control group (Table 4). Transformed into a risk ratio we can conclude that the likelihood of being a responder in the Aloe group increased by 94% relative to the control group (RR: 1.94; CI: 1.06–2.68).

**Table 4.** Summary of the binary generalized linear model to examine variables associated with treatment response in IBS-D.

Variable	Odds ratio (95% CI)	*p* value
Treatment (Aloe extract)	3.3 (1.08–9.98)	*0.036*
Gender (Female)	4.7 (1.29–16.81)	*0.019*
Baseline Symptom Severity	1.001 (0.99–1.007)	0.665

Significant *p* values are displayed in italics. CI, confidence interval; IBS-D, irritable bowel syndrome patients with diarrhoea.

### Aloe and control treatment were comparable in alleviating IBS symptoms in subgroups based on severity and psychological distress

We further explored the effect of the two treatments using the pooled data, in subgroups of IBS patients classified according to their baseline symptom severity^17^ and also in subgroups of patients with or without anxiety and depression.^19,21^ The IBS-SSS total score was similarly reduced in both treatment groups in the different IBS patient subgroups based on severity as seen in Table 5. The two treatments were also found to be comparable in reducing overall symptom severity in IBS patients with and without anxiety, as well as in IBS patients without depression (Table 6). When assessing IBS patients with depression (cut-off HADS depression score ⩾ 8), although including only a limited number of patients, a reduction in IBS-SSS total score was recorded in patients receiving Aloe treatment (*n* = 16, *p* = 0.001) but not control treatment (*n* = 16, *p* = 0.18), without difference between the groups (*p* = 0.16).

**Table 5.** Effects on symptoms in IBS subgroups based on severity (pooled data).

	Aloe group				Control group				*p* value between treatment groups^a^
	*n*	Baseline	End of treatment	*p* value within group	*n*	Baseline	End of treatment	*p* value within group	
IBS-SSS total score (mild)	9	150 ± 21	153 ± 83	0.93	20	137 ± 31	130 ± 65	0.66	0.75
IBS-SSS total score (moderate)	50	242 ± 41	206 ± 84	*0.003*	41	233 ± 36	207 ± 83	*0.04*	0.58
IBS-SSS total score (severe)	57	373 ± 46	309 ± 92	*<0.001*	46	365 ± 47	296 ± 97	*<0.001*	0.73

Data shown as mean ± SD. Significant *p* values are displayed in italics. IBS, irritable bowel syndrome; IBS-SSS, IBS Symptom Severity Score; Mild, IBS-SSS: 75 – 175; Moderate, IBS-SSS: 175 – 300; Severe, IBS-SSS: > 300.

a Based on change in IBS-SSS total score, comparing baseline vs end of treatment.

**Table 6.** Effects on symptoms in IBS subgroups based on psychological distress (pooled data).

	Aloe group				Control group				*p* value between treatment groups^a^
	n	Baseline	End of treatment	*p* value within group	n	Baseline	End of treatment	*p* value within group	
IBS-SSS total score (with anxiety^b^)	43	311 ± 99	268 ± 112	*0.003*	39	279 ± 107	216 ± 100	*<0.001*	0.29
IBS-SSS total score (without anxiety^b^)	73	292 ± 80	244 ± 100	*<0.001*	68	268 ± 91	240 ± 110	*0.006*	0.15
IBS-SSS total score (with depression^b^)	16	329 ± 81	262 ± 81	*0.001*	16	277 ± 120	248 ± 95	0.18	0.16
IBS-SSS total score (without depression^b^)	100	294 ± 88	251 ± 108	*<0.001*	91	271 ± 93	228 ± 109	*<0.001*	0.98

Data shown as mean ± SD. Significant *p* values are displayed in italics. HAD, Hospital Anxiety and Depression Scale; IBS, irritable bowel syndrome; IBS-SSS, IBS Symptom Severity Score.

a Based on change in IBS-SSS total score, comparing baseline vs end of treatment.

b Based on HAD. With anxiety or depression, HAD ⩾ 8; Without anxiety or depression, HAD ⩽ 8.

## Discussion

In this pooled subgroup analysis of two randomized controlled studies,^13,14^ treatment with Aloe extract provided significant improvement in overall symptom severity in IBS patients with diarrhoea, but not in other IBS subtypes (IBS-C and IBS-nonCnonD) based on predominant bowel habit. This was accompanied by higher frequency of responders in response to Aloe compared to the control treatment in the IBS-D group. The beneficial effect of Aloe extract seen in IBS-D patients was most pronounced for abdominal pain severity and pain frequency.

The current post hoc analysis demonstrated that Aloe extract has a significant effect in alleviating symptom severity in IBS-D patients. Although the effect of Aloe on stool frequency and stool consistency was comparable to the control treatment, indicating that bowel habits *per se* are not explicitly changed by Aloe, treatment with Aloe led to significant improvements in abdominal pain severity and frequency. These results are clinically relevant, not only since IBS-D patients account for approximately one-third of all IBS cases.^22^ Also, abdominal pain is a diagnostic requirement for IBS^23^ being one of the cardinal symptoms,^22^ and a significant predictor of overall IBS severity^24^ along with being the most common reason for patients to seek medical help.^25^ In addition, IBS-D patients report abdominal pain occurrence in more than 1 of 3 days, which may or may not be associated with their bowel movements.^26^

Aloe did not, however, show superiority over control treatment in alleviating IBS symptoms in other patient subgroups based on symptom severity or psychological distress. While Aloe showed a tendency of improved overall symptom severity in IBS patients with depression, this was based on relatively small patient numbers and could not contribute to conclusions. Although both men and women are affected by IBS, the prevalence is higher in women than in men.^27^ In our analysis, a greater proportion of responders in the IBS-D patient subtype were women, irrespective of the treatment. Gender-related variances in prevalence and pathophysiology^28^ as well as differences in response to treatment between men and women have been reported in IBS.^29^ While the underlying mechanisms for differences based on gender remains elusive, they may still contribute to the female-dominant treatment response seen in our study. Despite the randomization procedure in the two included studies, IBS-D patients receiving Aloe treatment in the pooled analyses had a higher baseline symptom severity, and the reduced symptom burden seen in this patient group could theoretically be due to regression towards the mean. However, results from the χ2 tests of homogeneity for response among the IBS-D patients, showed no evidence for response being linked to more severe symptoms in either of the two treatment groups, that is, all patients despite their baseline symptom severity could likely respond to the two treatments. The generalized linear model corrected for baseline variables further established the Aloe treatment effect on symptoms among IBS-D patients and confirm the robustness of our results.

The potential mechanism of action of Aloe remains elusive. Traditionally, Aloe has been used to treat symptoms of constipation owing to laxative properties linked to the hydroxyanthracene derivatives or anthraquinones.^30^ This component of Aloe is, however, also associated with safety concerns when ingested in excess amounts.^9^ The Aloe extract used in our studies is a purified freeze dried inner leaf gel extract containing only traces (less than 0.1 ppm) of aloin (anthraquinone) and an enriched polysaccharide content. While the symptom reducing effect seen by Aloe extract in the IBS-D group in our study cannot be due to aloin content, it may be implicated to one or more of the 75 biologically active components present in Aloe gel, with potential wound healing, anti-oxidant, anti-inflammatory, prebiotic properties and pain relief properties.^9,10,31–33^ In our study, Aloe improved pain severity and frequency among IBD-D patients but not the other IBS subtypes based on bowel habit. The subtype based variance in abdominal pain may reflect differences in underlying pathophysiological mechanisms leading to different responses to therapeutic agents.^34^ Indeed, distinct gut microenvironment linked to intestinal bowel habits may be a potential factor explaining differences in clinical response to different treatments.^35^ In line with this, we recently reported that Aloe extract has potential prebiotic effect, showing a relationship between treatment response and gut microbiota-metabolite profiles,^13^ suggesting another possible mechanism of action of Aloe treatment. Considering the complex and multifactorial pathophysiology of IBS, treatment with Aloe extract composed of numerous bioactive components, implicated to its diverse therapeutic effects, may have other advantages compared to pharmacological agents which have more precise modes of action.

To our knowledge, this is the first study supporting the treatment effect of Aloe extract in IBS patients with diarrhoea, seen as an improvement in overall symptom severity, in particular pain severity and pain frequency. However, despite the promising results, a limitation of this pooled analysis includes the post hoc nature of data analysis. The formulation of the treatment tablets used in our controlled studies is another weakness, with both the Aloe treatment and control treatment tablets containing inulin at different doses. While initially regarded as a placebo in our pilot study,^14^ inulin has more recently been shown to have beneficial effects on healthy subjects^36–38^ and should hence be regarded and used as control treatment.^13^ Although the control treatment in our studies consisted only of a low dose of inulin, approximately 1/4 of the dose previously shown to have effect on gut microbiota, with the Aloe treatment tablet containing an even lower dose, we cannot entirely rule out that inulin may have influenced the gut microbiota. Nevertheless, since both treatment tablets in our studies contained inulin, and the beneficial treatment response in IBS patients with diarrhoea was seen only in the Aloe treatment group, the treatment effect can safely be associated with Aloe extract.

The treatment of patients with severe IBS symptoms such as abdominal pain is challenging and despite the numerous reasonably effective interventions available for IBS-D, including diet alterations and pharmacological treatments, patients continue to have a substantial symptom burden and impairments to quality of life.^22,39^ Thus, alternative therapy options like Aloe extract, offering an effective improvement in the overall symptom severity of IBS-D patients, without significant side effects makes our results of worthy focus.

In conclusion, this pooled analysis of two controlled studies indicates that treatment with Aloe extract provides clinically meaningful improvement in symptom severity in patients with IBS-D, with reduction in abdominal pain severity and pain frequency, the key symptoms in IBS. Therefore, Aloe extract is a safe and effective treatment option in this patient group.

## Supplemental Material

sj-docx-1-tag-10.1177_17562848211048133 – Supplemental material for Aloe barbadensis Mill. extract improves symptoms in IBS patients with diarrhoea: post hoc analysis of two randomized double-blind controlled studies Supplemental material, sj-docx-1-tag-10.1177_17562848211048133 for Aloe barbadensis Mill. extract improves symptoms in IBS patients with diarrhoea: post hoc analysis of two randomized double-blind controlled studies by Bani Ahluwalia, Maria K. Magnusson, Lena Böhn, Stine Störsrud, Fredrik Larsson, Lena Öhman and Magnus Simrén in Therapeutic Advances in Gastroenterology
