# Supplementary material for: Aloe barbadensis Mill. extract improves symptoms in IBS patients with diarrhoea: post hoc analysis of two randomized double-blind controlled studies
Source: Ther Adv Gastroenterol. 2021 Oct 8;14:17562848211048133. doi: 10.1177/17562848211048133 (PMC8504273; doi:10.1177/17562848211048133)
Supplement: sj-docx-1-tag-10.1177_17562848211048133 – Supplemental material for Aloe barbadensis Mill. extract improves symptoms in IBS patients with diarrhoea: post hoc analysis of two randomized double-blind controlled studies [file sj-docx-1-tag-10.1177_17562848211048133.docx]

**Supplement Material**

**Data S1 Methods**

**Table S1** Study patients in different phases of Study A and Study B.

**Methods**

***Symptom Assessment Questionnaires***

*Bristol Stool Form (BSF) scale*

The BSF scale^1^ was used to record stool frequency (number of stools per day), and mean stool consistency on a 7-point scale. The BSF scale was also used to determine the IBS subgroups, that is, IBS with constipation (IBS-C), IBS with diarrhoea (IBS-D), mixed IBS with loose and hard stools (mixed bowel habits) (IBS-M) or unsubtyped IBS (IBS-U), where the two latter groups were combined into one group (IBS-nonCnonD).

*IBS-Symptom Severity Scoring (IBS-SSS) System*

The IBS-SSS was used to assess the severity of IBS symptoms. This questionnaire uses visual analogue scales (VAS) (0-100mm); the overall score is calculated from five items: pain severity, pain frequency, bloating severity, bowel habit dissatisfaction and influence of IBS on life in general or life interference, with a range of 0 to 500. This questionnaire allows the classification of patients into the following severity subgroups: mild (≤174 points), moderate (175 – 300), severe (>300).^2^

*Hospital Anxiety and Depression Scale (HADS)*

The HADS is a 14-item questionnaire used to measure the severity of anxiety and depression on 2 subscales with 7 items each. Each item is scored using a 4-point Likert scale, between 0 and 3, with higher scores indicating more severe symptoms. Total score range per subscale goes from 0 to 21 points and comprises three different subgroups.^2, 3^ The HAD subscale classifies patients without reported anxiety or depression (HAD score <8); borderline anxiety or depression (HAD score 8 - 10) or clinically significant anxiety or depression (HAD score ≥11). In this study a HAD score ≥8 was considered as clinically significant anxiety or depression, while a HAD score <8 was considered as absence of anxiety or depression.

­­­­­­­­­­­­­­­­­­­

**Table S1.** Study patients in different phases of Study A and Study B.

|  | **Study A** | | **Study B** | |
| --- | --- | --- | --- | --- |
|  | Aloe group | Control group | Aloe group | Control group |
| Randomised | 33 | 35 | 91 | 82 |
| Drop Outs | 1 | 4 | 7 | 6 |
| Completed Study | 32 | 31 | 84 | 76 |

**References**

1. Longstreth GF, Thompson WG, Chey WD, et al. Functional bowel disorders. *Gastroenterology* 2006; 130: 1480-1491. DOI: 10.1053/j.gastro.2005.11.061.

2. Bjelland I, Dahl AA, Haug TT, et al. The validity of the Hospital Anxiety and Depression Scale. An updated literature review. *J Psychosom Res* 2002; 52: 69-77. 2002/02/08. DOI: 10.1016/s0022-3999(01)00296-3.

3. Zigmond AS and Snaith RP. The hospital anxiety and depression scale. *Acta Psychiatr Scand* 1983; 67: 361-370.
